# Plant Part–Specific Differences in Ashwagandha (*Withania somnifera*): A Comparative Evaluation of Root and Leaf Evidence for Safety and Regulation

**DOI:** 10.1002/fsn3.72364

**Published:** 2026-10-05

**Authors:** Abhijit Dutta, Jang Bahadur Gupta

**Affiliations:** ^1^ Ministry of Ayush (Government of India) New Delhi India; ^2^ World Ashwagandha Council New Delhi India

**Keywords:** ashwagandha, safety, *Withania somnifera*, withanolides

## Abstract

Ashwagandha ( *Withania somnifera* ) is a key herbal product traditionally used as a root‐based rejuvenative. Leaf and whole‐plant preparations have also become increasingly available, for which dedicated safety characterization remains limited. European risk assessments and the Danish ban have raised safety concerns for “ashwagandha” without consistently distinguishing plant parts. We conducted a structured narrative review with a regulatory focus, integrating traditional sources, phytochemical and pharmacologic studies, human clinical trials, pharmacovigilance data, quality/adulteration investigations, and national and international risk assessments. Electronic databases and gray literature sources (including AYUSH, DTU, RIVM, and FSA) were searched through 31 March 2026, and evidence was synthesized qualitatively along the axes of traditional use, phytochemistry, efficacy, safety, quality and regulation, explicitly contrasting root and leaf. Root consistently shows a profile dominated by withanolide A, D, and glycol‐withanolides, whereas leaf is richer in withaferin A and related electrophilic withanolides. Multiple randomized controlled trials and a 12‐month study of standardized root extracts demonstrate clinically relevant reductions in stress, anxiety, and insomnia, with generally favorable safety and no reproducible serious organ toxicity. Human data for leaf are sparse and confounded, while preclinical leaf studies emphasize the high withaferin A content and anticancer and immunomodulatory effects and theoretical toxicity concerns. European risk opinions have extrapolated mixed plant‐part data to all ashwagandha preparations, even as analytical surveys document adulteration of root materials with leaves and stems. Current evidence supports greater confidence in authenticated, standardized root preparations for systemic use, while evidence for leaf remains limited and requires indication‐specific development and dedicated safety evaluation. These findings support plant‐part specificity in regulatory frameworks, including analytical authentication to detect leaf adulteration, plant‐part‐ and product‐specific risk assessment, and re‐assessment of plant‐part‐agnostic safety conclusions as higher‐quality evidence emerges. Future research should prioritize comparative trials of root versus leaf and whole‐plant extracts and long‐term safety studies of standardized leaf preparations.

## Graphical abstract

This review contrasts the root and leaf of *Withania somnifera* (ashwagandha) across phytochemistry, clinical evidence, and regulatory context. Root preparations are dominated by withanolide A, D, and glyco‐withanolides, with multiple randomized controlled trials supporting reductions in stress, anxiety, and insomnia alongside a favorable safety profile. Leaf preparations are rich in electrophilic withaferin A, with sparse human data and theoretical toxicity concerns. Regulatory frameworks have conflated plant parts, compounded by documented adulteration. Recommendations include plant‐part‐specific risk assessment, analytical authentication, and dedicated long‐term safety evaluation of leaf preparations.

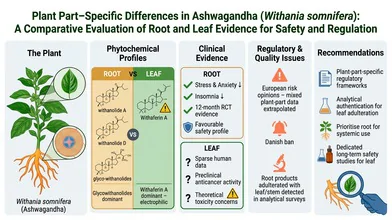

**Abbreviations**
AI: artificial intelligence
ALP: alkaline phosphatase
ALT: alanine aminotransferase
ANSES: French Agency for Food, Environmental and Occupational Health & Safety
AST: aspartate aminotransferase
BAPP: Botanical Adulterants Prevention Program
BfR: German Federal Institute for Risk Assessment — Bundesinstitut für Risikobewertung
DILI: drug‐induced liver injury
DNA: deoxyribonucleic acid
DTU: Technical University of Denmark — Danmarks Tekniske Universitet
EMA: European Medicines Agency
EU: European Union
FSA: UK Food Standards Agency
GABA: gamma‐aminobutyric acid
GLP: good laboratory practice
GMP: good manufacturing practice
HILI: herb‐induced liver injury
HPA: hypothalamic‐–pituitary‐–adrenal axis
HPLC: high‐performance liquid chromatography
HPTLC: high‐performance thin‐layer chromatography
NFκB: nuclear factor kappa B
NLP: natural language processing
Nrf2: nuclear factor erythroid 2‐related factor 2
QSAR: quantitative structure–activity relationship
RCT: randomized controlled trial
RIVM: National Institute for Public Health and the Environment, Netherlands—Rijksinstituut voor Volksgezondheid en Milieu
RUCAM: Roussel Uclaf Causality Assessment Method
STAT3: signal transducer and activator of transcription 3
UHPLC–MS/MS: ultra‐high‐performance liquid chromatography–tandem mass spectrometry
WHO: World Health Organization

## 1 Introduction

Ashwagandha ( *Withania somnifera* L. Dunal) is a key herbal ingredient, traditionally known as a rejuvenative tonic to enhance strength, vitality, cognition, and resilience to stress (Mikulska et al. 2023). Classical Ayurvedic formulations predominantly employ the root as the officinal part, and this preference has been mirrored in contemporary pharmacological and clinical research, where standardized root extracts are widely investigated for adaptogenic, anxiolytic, neuroprotective, and cardiometabolic benefits (Mikulska et al. 2023; MoA 2024a). In parallel, global consumer demand for ashwagandha‐containing supplements has increased sharply, raising concerns around sustainable raw material supply, quality assurance, and the need for evidence‐based specification of plant part(s) in regulatory frameworks (MoA 2024a; Khabiya et al. 2024; Patwardhan et al. 2024).

Despite the root‐centric paradigm, the leaf of *Withania somnifera* (WS) has a history of folkloric and regional use in India for conditions such as pain, inflammatory swellings, skin disorders, and metabolic ailments, administered both topically and orally (Bhosale et al. 2022; Beladiya et al. 2024; Singirala et al. 2025). Recent phytochemical and pharmacological investigations demonstrate that ashwagandha leaves contain a rich spectrum of withanolides and other bioactives, including withaferin A and withanone, which exhibit anticancer, anti‐inflammatory, neuroprotective, and metabolic effects in preclinical models (Mikulska et al. 2023; Bhosale et al. 2022; Singirala et al. 2025). These findings have prompted the proposition that leaves, being renewable and easier to harvest without compr the entire plant, could serve as an alternative or complementary therapeutic part to roots, potentially supporting sustainability and supply security for industry and health systems (Bhosale et al. 2022; MoA 2024b).

The emerging regulatory landscape in Europe has brought these questions into sharper focus. In 2020, the Danish National Food Institute (DTU) published a risk assessment on *WS*, which led the Danish Veterinary and Food Administration (DVFA) to decide to ban *WS* in food supplements, largely on the basis that a safe lower intake level could not be established, without clearly differentiating between root and aerial parts (Patwardhan et al. 2024; Berra et al. 2024; Brendler et al., n.d.; Chittiboyina and Khan 2024; Morandi 2024). Subsequent national evaluations, including the French evaluation (ANSES 2024) and the German BfR report (BfR 2024), have echoed concerns about potential hepatotoxicity, thyroid effects, and reproductive toxicity, leading to precautionary restrictions or advisories in several EU member states, even as some authorities (e.g., Poland and Hungary) explicitly allow root but not leaf preparations (ANSES 2024; FSA 2024; Tsoulli et al. 2025).

Global regulators and expert groups broadly classify *WS* as a herbal dietary or traditional medicine ingredient but increasingly emphasize the need to distinguish root‐only preparations from those containing aerial parts, including leaves, because of differing withanolide profiles and toxicological signals (Berra et al. 2024; ANSES 2024). Concerns about economic adulteration, where products labeled as “root” or “root‐only” are partially substituted with cheaper leaves and stems, have prompted initiatives such as the US Botanical Adulterants Prevention Program's laboratory guidance document, which documents frequent detection of leaf material in commercial “root” extracts and warns that total‐withanolides tests are insufficient to detect such adulteration (Singh et al. 2018; Amritha et al. 2020; Kumar et al. 2026). These developments frame *WS* not merely as a generic “herb” but as a complex, multi‐part medicinal plant in which the choice and authentication of plant part have direct implications for safety assessment, regulatory status, and clinical acceptability.

However, critical questions remain regarding the comparative efficacy, safety, and regulatory acceptability of *WS* leaf in relation to the root, especially for oral, long‐term use in stress‐related and chronic conditions that currently rely on root‐based evidence (MoA 2024a; Berra et al. 2024; Singh et al. 2011). While narrative and scoping reviews have catalogued a broad range of health‐promoting activities of *WS* across plant parts, there is limited systematic appraisal along the axis of “root versus leaf” that integrates traditional textual evidence, modern phytochemistry, preclinical pharmacology, human data, and regulatory perspectives (Bhosale et al. 2022; Singirala et al. 2025; Berra et al. 2024). Against this background, this structured narrative review examined comparative evidence on *WS* root versus leaf regarding efficacy, safety, quality, and regulatory status.

## 2 Methods

Electronic searches were conducted in PubMed, Scopus, and Web of Science, supplemented by Google Scholar, through 31 March 2026. The full electronic search strategy for each database, including field tags and Boolean strings, is provided in Table S1; gray‐literature and official sources are listed in Table S2. Initially, a total of 1514 records were retrieved (1497 from databases and 17 from other sources); however, finally 56 records were ultimately included in the review. The detailed study selection process is provided in Figure 1.

**FIGURE 1:**
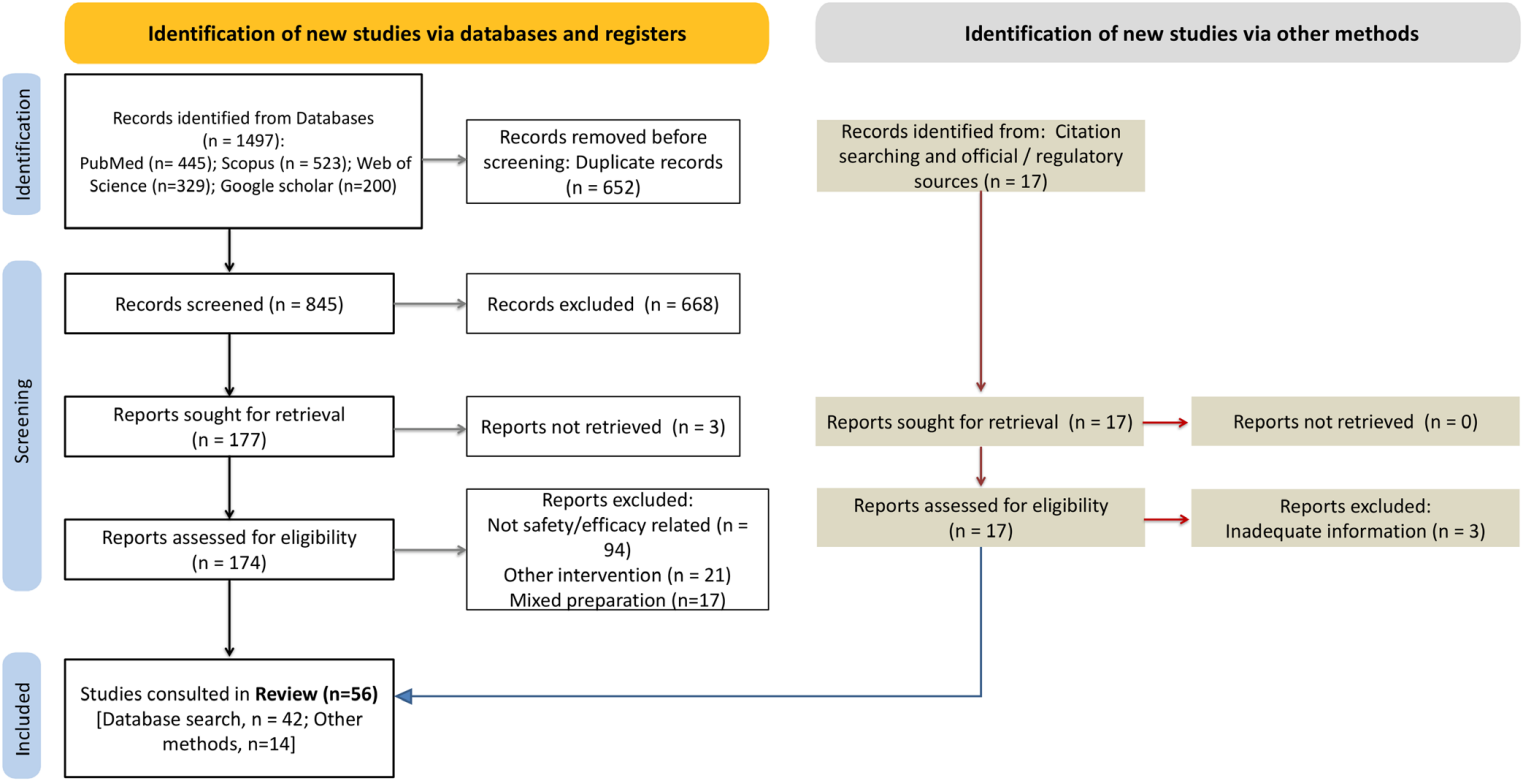
Study flow diagram.

Data were narratively synthesized, structured around four themes: (i) traditional and ethnomedicinal uses of root versus leaf, (ii) phytochemical and pharmacological differences, (iii) clinical and safety signals by plant part, and (iv) regulatory and quality perspectives, with particular attention to European assessments (notably DTU and RIVM) and critiques of their methodology.

## 3 Results

### 3.1 Phytochemical Considerations

The phytochemical composition of *WS* is dominated by steroidal lactones known as withanolides, together with alkaloids, flavonoids, phenolic compounds, and sitoindosides, but the qualitative and quantitative profile of these constituents differs markedly between root and leaf (Mikulska et al. 2023; Chaurasiya et al. 2008; Kumar et al. 2023, 2024). Comparative HPLC and UHPLC–MS/MS studies show that leaves typically accumulate higher levels of withaferin A and related epoxide‐bearing withanolides, whereas roots are relatively enriched in withanolide A, withanolide D, and certain glycowithanolides that are often selected as marker compounds in pharmacopoeial monographs and standardized commercial root extracts (Chaurasiya et al. 2008; Singh et al. 2025). In varietal and tissue‐specific analyses, withaferin A content has been reported to be highest in field‐grown leaves of chemotypes such as Poshita, while withanolide A predominates in roots across multiple chemotypes, underscoring a consistent leaf–root gradient in the ratio of more cytotoxic versus “tonic‐type” withanolides (Chaurasiya et al. 2008; Thorat et al. 2022; Zellner et al., n.d.). Leaf‐focused reviews further catalogue a broader array of additional withanolides (e.g., 27‐deoxywithaferin A, 27‐hydroxywithanolide B, and 7‐hydroxywithanolide) and minor constituents that contribute to potent anti‐inflammatory and anticancer activities, but also raise theoretical safety concerns when high‐withaferin A leaf material is substituted into preparations developed and evaluated as “root‐only” (Bhosale et al. 2022; Singirala et al. 2025; Zellner et al., n.d.). By contrast, roots, while containing withaferin A in lower amounts, provide a withanolide spectrum that has been more extensively standardized and clinically characterized, and this root‐biased evidence base underpins pharmacopeial standards and most global regulatory discussions that currently treat ashwagandha as a predominantly root‐derived herb, even though analytical surveys increasingly detect leaf‐derived withanolide fingerprints in products labeled as “root” extracts, signaling adulteration and complicating the interpretation of safety and efficacy data (Bhosale et al. 2022; Chaurasiya et al. 2008; Singh et al. 2025).

### 3.2 Traditional and Ethnomedicinal Uses of Root and Leaf

Across Ayurvedic classical texts, ashwagandha is primarily described as a root‐based Rasayana, prescribed as a medhya and balya tonic for conditions such as stress, debility, emaciation, insomnia, and neuromuscular disorders, often administered as churna (powder) with milk or ghee (Bhosale et al. 2022; Singirala et al. 2025; Singh et al. 2011). Modern compilations of traditional practice also document root use in arthritis, rheumatic pain, male reproductive dysfunction, and as an adjuvant in chronic infections, mirroring its broad Rasayana positioning in classical literature (Mikulska et al. 2023; Dar et al. 2015; Guo and Rezaei 2024).

In contrast, the leaf of *W. somnifera* , though only sparsely mentioned in classical Ayurvedic compendia, features prominently in regional and tribal ethnomedicine in India where it is applied locally as pastes, poultices, or decoctions for painful swellings, wounds, boils, scorpion stings, and dental pain, and is sometimes ingested for uterine displacement, fever, and metabolic complaints (Bhosale et al. 2022; Wadhwa et al. 2016). Ethnopharmacological surveys and pharmacologic overviews further note internal use of leaf or mixed leaf–root preparations in diabetes, asthma, and hypertension, explained by preclinical data showing that leaf extracts can exert strong antihyperglycaemic and insulin‐sensitizing effects in experimental models (Bhosale et al. 2022; Dar et al. 2015; Wadhwa et al. 2016).

### 3.3 Preclinical Pharmacology

The preclinical pharmacology of *WS* reflects both shared and distinct activities of root and leaf, with most in vivo data generated on root or root‐standardized extracts and a growing, but still largely experimental, body of evidence for leaf‐derived preparations (Chaurasiya et al. 2008; Dar et al. 2015; Wadhwa et al. 2016). Root extracts demonstrate robust anti‐stress, anxiolytic, anti‐inflammatory, anti‐arthritic, cardioprotective, and neuroprotective effects across rodent models, often linked to modulation of the HPA axis, GABAergic and serotonergic signaling, NF‐κB, and Nrf2 pathways, and pro‐inflammatory cytokines, alongside anticancer and anti‐adipogenic actions at higher doses (MoA 2024a, 2024b; Dar et al. 2015; Bashir et al. 2023).

By contrast, leaf extracts, which are richer in withaferin A and certain electrophilic withanolides, show potent anticancer, immunomodulatory, and neuroprotective effects in vitro and in selected in vivo models, including selective killing of tumor cells, inhibition of NF‐κB and STAT3 signaling, and protection of neuronal and glial cells against oxidative and glutamate‐induced injury (Wadhwa et al. 2013; Widodo et al. 2008). In some comparative experiments, leaf or aerial‐part extracts match or exceed root extracts in endpoints such as antioxidant capacity, antitumour activity, and metabolic modulation; however, these studies often use higher withaferin A exposures and short durations, leaving uncertainties about translational safety margins for chronic oral use relative to established root‐based regimens (Kumar et al. 2023; Dar et al. 2015). Overall, preclinical data support the pharmacologic plausibility of both root and leaf as bioactive parts of *WS*, but the weight of in vivo, dose‐ranging, and mechanistic evidence currently favors root extracts for systemic, long‐term administration, with leaf data remaining more exploratory and focused on oncology and neuroprotection.

### 3.4 Clinical Evidence

Nearly all controlled human evidence for ashwagandha derives from root extracts, and leaf‐specific or mixed‐preparation human data are minimal (MoA 2024a, 2024b; Kumar et al. 2026; Lucius 2025; Lopresti and Smith 2021; Wiciński et al. 2025). Multiple randomized, double‐blind, placebo‐controlled trials of root‐only extracts (typically 240–600 mg/day) in adults with stress, anxiety, or poor sleep show consistent improvements in perceived stress scores, anxiety scales, sleep quality indices, and cortisol, with generally favorable tolerability and no clinically meaningful changes in routine biochemistry or hematology over 6–12 weeks (Akhgarjand et al. 2022; Arumugam et al. 2024; Cheah et al. 2021; Della Porta et al. 2023; Fatima et al. 2024; Gómez Afonso et al. 2023). Longer‐term data, including a 12‐month open‐label study, report an absence of serious adverse drug reactions and no signal for cumulative hepatic, renal, thyroid, or hematologic toxicity with properly standardized root extracts (KSM‐66; 600 mg/day) administered orally in adults (Gómez Afonso et al. 2023). Moreover, a systematic review incorporating 30 clinical trials of *root* preparations across multiple indications, including chronic stress, anxiety, insomnia, cognitive enhancement, rheumatoid arthritis, type 2 diabetes, and male infertility, reported no serious adverse events and no clinically meaningful changes in hematological, biochemical, or vital parameters across studies (Tandon and Yadav 2020).

A limited but growing body of clinical evidence has examined either standalone leaf extracts or combined root‐and‐leaf formulations. A Phase I open‐label clinical trial investigated a pharmaceutical‐grade leaf extract (RH324) in patients with advanced non‐small cell lung cancer, reporting tolerability with no serious treatment‐related adverse events and preliminary evidence of disease stabilization over a 28‐day treatment period (Heo et al. 2026). A few RCTs have reported the efficacy of combined root‐and‐leaf aqueous extracts in adults with chronic stress, at doses ranging from 125 to 500 mg daily over eight weeks (Pandit et al. 2024; Auddy et al. 2008). Additionally, the STAR trial reported improvements in muscle strength, training adaptation, and recovery markers following supplementation with a standardized root‐and‐leaf extract in healthy adults engaged in resistance training (Ziegenfuss et al. 2018).

Pharmacovigilance data show rare but occasionally severe liver injury in users of commercial ashwagandha products (Bokan et al. 2023) compiled 12 published cases across 8 publications, and in these reports, the product's plant part was not analytically characterized (unnamed, bought online, or just labeled “ashwagandha capsules”). RUCAM causality scores ranged only from “possible” to “probable,” none reaching “definite,” reflecting both the idiosyncratic nature of the injury and the lack of product‐level data. Severity ranged from self‐limiting injury that resolved after stopping the product to one case of acute liver failure requiring transplant.

Notably, (Bokan et al. 2023) hypothesize that undeclared leaf adulteration, which contains substantially higher levels of withanone and withaferin A than root, may contribute to hepatotoxicity, with differential withanolide content providing a plausible mechanistic basis. The only clinical evidence definitively linking hepatotoxicity to a leaf‐derived constituent is the Phase I trial of pharmaceutical‐grade leaf extract (RH324) by (Pires et al. 2020), where dose‐dependent liver enzyme elevations occurred in five of 11 osteosarcoma patients, a population and dose context distinct from typical supplement users.

### 3.5 Quality and Regulatory Perspectives

The quality and regulatory landscape around *WS* is increasingly shaped by concerns over plant‐part specificity and adulteration of root materials with leaves and stems. Pharmacopoeial standards and national monographs [such as the Ayurvedic Pharmacopeia of India and the European Medicines Agency (EMA)] generally define ashwagandha as the dried root or root extract, with identity tests, minimum withanolide content, and limits for contaminants anchored in root material and its characteristic organoleptic profile (MoA 2024b; EMA 2011). However, enforcement testing and independent investigations show that a proportion of commercial products labeled as “ashwagandha root” or “root‐only extract” actually contain undeclared aerial parts, particularly leaves, which are financially cheaper and richer in withaferin A, thereby altering both phytochemical composition and risk profile (Singirala et al. 2025; Singh et al. 2018; Dutta et al. 2019).

The Botanical Adulterants Prevention Program (BAPP) has issued both a bulletin and a detailed Laboratory Guidance Document on Ashwagandha, documenting cases where powdered leaves and stems were blended into root powders or extracts and warning that assays based solely on “total withanolides” cannot reliably distinguish root from leaf because withanolides occur throughout the plant (Singirala et al. 2025; Singh et al. 2018). These documents recommend orthogonal analytical approaches, such as HPTLC/HPLC fingerprinting, targeted quantification of withaferin A and quercetin‐related metabolites, and DNA‐based tools to authenticate root‐only materials and detect adulteration, and explicitly link mislabeling with compromised safety assessment, given that much of the toxicological signal in some European risk evaluations derives from studies on leaf or whole‐plant preparations rather than on authenticated root (MoA 2024b; Berra et al. 2024; ANSES 2024). In this context, global regulators broadly categorize ashwagandha as a “herbal” ingredient but are increasingly alert to the fact that even products claimed as root‐based may, in practice, be whole‐plant or leaf‐adulterated, which complicates causality assessment in hepatotoxicity reports and underpins calls in Europe, the UK, and the Nordics for clearer specification, testing, and labelling of plant part as a critical quality attribute (ANSES 2024; FSA 2024; Tsoulli et al. 2025; Technical University of Denmark (DTU) 2020).

The regulatory responses and risk perspectives on *WS* over the last decade illustrate a widening divergence between jurisdictions that prioritize long‐standing root‐based use and those applying highly precautionary interpretations of mixed or poorly characterized datasets, often without distinguishing clearly between root and aerial parts (MoA 2024a; Morandi 2024). In Denmark, a 2020 literature‐based risk assessment by the Technical University of Denmark (DTU) for the Danish Veterinary and Food Administration (DVFA) concluded that a safe intake level for ashwagandha in food supplements could not be established, citing potential effects on thyroid and sex hormones and possible abortifacient properties; this opinion led to an outright national ban on ashwagandha‐containing supplements from 2023 (MoA 2024a, 2024b; Patwardhan et al. 2024; Morandi 2024). Independent academic critiques have argued that the DTU report over‐weighted limited animal and case‐report data, conflated root with leaf and whole‐plant preparations, relied on low‐quality sources, and failed to account for the broader clinical safety dataset on standardized root extracts, prompting calls from Indian authorities and international experts for its reconsideration as an example of regulation that “ignores evidence” (MoA 2024a; Patwardhan et al. 2024; Chittiboyina and Khan 2024).

Following Denmark, several European and Nordic institutions, including the Dutch RIVM (RIVM 2024), Germany's BfR (BfR 2024), France's ANSES (ANSES 2024), and the UK Food Standards Agency (FSA 2024) have undertaken or commissioned risk assessments that highlight signals for hepatotoxicity, thyroid perturbation, and reproductive concerns, and in some cases advise against use in vulnerable groups such as pregnant or breastfeeding women, minors, and people with pre‐existing liver, thyroid, or cardiac disease. Notably, these assessments frequently acknowledge that much of the adverse‐event literature involves products of uncertain composition and plant part, while clinical trials on authenticated root extracts have not demonstrated comparable risks at typical doses; nevertheless, the combined precautionary stance has led to bans (e.g., Denmark), de facto discouragement or ““do not use” advice (e.g., the RIVM opinion), and proposals for stricter labeling and maximum daily doses in several EU member states (Berra et al. 2024; RIVM 2024). In parallel, regulators and expert groups worldwide, including AYUSH (MoA 2024a, 2024b), and authors of recent regulatory reviews (Berra et al. 2024), have increasingly stressed that future risk assessments should differentiate clearly between root and aerial parts, avoid extrapolating leaf or whole‐plant toxicity data to root‐only products, and explicitly consider the possible confounding role of leaf adulteration in European hepatotoxicity case clusters (Bokan et al. 2023) if regulatory decisions are to remain proportionate, evidence‐based, and respectful of both traditional practice and modern pharmacology (MoA 2024b; Berra et al. 2024; Chittiboyina and Khan 2024; NIDDK 2012).

## 4 Discussion

The findings of this review indicate that ashwagandha (*WS*) root and leaf cannot be treated as interchangeable entities from either a pharmacologic or a regulatory standpoint, and that a plant‐part‐agnostic “whole‐plant” concept is insufficiently supported by current evidence. It should be emphasized at the outset that the case for plant‐part specificity rests on the marked asymmetry in evidence maturity between root and leaf, together with documented adulteration of root products, rather than on any demonstration that leaf is inherently unsafe; the current leaf evidence is best characterized as insufficient for chronic systemic use rather than as evidence of harm. Traditional Ayurvedic sources and ethnomedicinal surveys converge on the root as the principal Rasayana part for internal use, whereas the leaf is used more locally, often topically or in indication‐specific ways, which already suggests a historical differentiation of expected benefit–risk profiles. Modern phytochemical and tissue‐profiling studies consistently show that leaves are relatively enriched in withaferin A and other electrophilic withanolides, while roots contain higher proportions of withanolide A, withanolide D, and glycowithanolides that have become the de facto quality markers in pharmacopoeial standards and commercial root extracts. This biochemical gradient aligns with the observation that most preclinical and virtually all robust clinical data on systemic, long‐term administration involve authenticated root extracts, which demonstrate consistent benefits in stress, anxiety, sleep disturbance, and related endpoints, together with a favorable safety profile up to 12 months and no reproducible signal for serious hepatic, renal, hematologic, or reproductive toxicity at studied doses. Moreover, a systematic review incorporating 30 clinical trials also informed the safety of the ashwagandha root extract (Tandon and Yadav 2020). A recently published computational safety evaluation employing natural language processing (NLP)‐based meta‐analysis of 1396 PubMed‐indexed publications and QSAR‐based molecular toxicity prediction further reinforces this pattern. Ronen et al. reported that none of the 79 molecules predicted to be present in the *WS* root were flagged for liver toxicity. These computational findings are consistent with the clinical and preclinical evidence reviewed above and provide additional mechanistic rationale for plant‐part differentiation in safety assessment. In contrast, evidence for leaf remains dominated by in vitro and short‐term in vivo models in oncology, immunology, and neuroprotection, using withaferin A–rich exposures and providing insufficient basis for defining safe chronic oral doses in humans.

The traditional preference for *WS* roots in classical Ayurvedic practice reflects the therapeutic priorities of that system, wherein ashwagandha was principally classified as a *rasayana*, a rejuvenating agent intended to promote vitality, enhance stress resilience, and improve overall quality of life in healthy or subclinically stressed (Lopresti and Smith 2021; Arumugam et al. 2024; Kaul and Wadhwa 2024). Within this framework, the root was the logical plant part of choice, as its withanolide profile, characterized by relatively higher proportions of withanolide A and lower concentrations of withaferin A, aligns well with adaptogenic and anabolic activity at physiological doses (Singh et al. 2011; Kaul and Wadhwa 2017). Emerging preclinical and early clinical evidence indicates that *WS* leaves, which contain substantially higher concentrations of withaferin A compared with roots, exhibit significant cytotoxic, pro‐apoptotic, and anti‐proliferative activity across multiple cancer cell lines, positioning leaf‐derived compounds as promising candidates for drug discovery and pharmaceutical development (Wadhwa et al. 2013; Kaul and Wadhwa 2024). Accordingly, a scientifically balanced perspective must distinguish between the *therapeutic repositioning* of leaf‐derived bioactives within a pharmaceutical drug‐discovery pipeline, which merits active investigation, and the unregulated addition of leaf material to commercial nutraceutical products, where safety thresholds remain undefined and consumer populations are heterogeneous. The present manuscript does not discount the pharmacological significance of *WS* leaves; rather, it advocates for a regulatory framework that is commensurate with their biological potency.

From a benefit–risk perspective, the root benefits from several merits: (i) long, relatively coherent traditional use; (ii) defined pharmacopeial specifications anchored to root morphology and root‐specific withanolide patterns; and (iii) a comparatively mature clinical dataset and emerging modeling data pointing to a wide therapeutic margin within studied dose ranges.

Its principal demerits are extrinsic: whole‐root harvesting is less sustainable than leaf harvesting, and rising global demand creates economic incentives for adulteration of root materials with leaves and stems, which in turn confounds both pharmacovigilance and regulatory signal detection. The leaf, by contrast, offers pharmacologic advantages and regulatory challenges in equal measure. Its high withaferin A content underpins potent anticancer, anti‐inflammatory, and neuroprotective effects in experimental systems and argues for targeted drug‐development pathways for defined indications. Yet the same profile raises a higher a priori toxicity concern for chronic systemic use, and human data remain sparse, fragmented, and often confounded by mixed root–leaf formulations and inadequate chemical characterization.

These scientific nuances intersect directly with regulatory practice. The Danish DTU risk assessment, which underpinned the national ban on ashwagandha in food supplements, explicitly declined to establish a safe intake level for “ashwagandha” based on a heterogeneous body of evidence that largely failed to distinguish root from aerial parts and relied heavily on low‐quality or non‐GLP animal studies and case reports (Patwardhan et al. 2024; Chittiboyina and Khan 2024; Morandi 2024). Subsequent critiques from AYUSH (MoA 2024a) and independent academic authors (Patwardhan et al. 2024; Chittiboyina and Khan 2024; Morandi 2024) have argued that the DTU approach conflated leaf/whole‐plant toxicity with root‐only experience and overlooked the weight of clinical evidence, thereby illustrating how non‐differentiated plant‐part treatment can lead to over‐precautionary regulation. Other European risk assessments (RIVM, BfR, ANSES, FSA) adopt a more nuanced tone but still aggregate adverse‐event signals from products of uncertain composition with findings from leaf or whole‐plant studies, and then generalize their conclusions to “ashwagandha” as a category, even while acknowledging that clinically studied root extracts have not demonstrated comparable risks at therapeutic doses (ANSES 2024; BfR 2024; Tsoulli et al. 2025; RIVM 2024). In parallel, the Botanical Adulterants Prevention Program has documented systematic adulteration of root materials with leaves and stems and has shown that simple “total withanolide” assays lack discriminatory power, necessitating orthogonal methods that combine chromatographic fingerprints, withaferin A ratios, and DNA approaches for reliable plant‐part authentication (Bhosale et al. 2022; Amritha et al. 2020; Chaurasiya et al. 2008; Kumar et al. 2023; Widodo et al. 2008). These findings imply that some hepatotoxicity and endocrine‐signal clusters in Europe may, in fact, reflect exposure to unlabeled leaf or whole‐plant material in products marketed as “root”, underscoring the regulatory importance of quality and authenticity rather than plant identity alone (MoA 2024a; NIDDK 2012). A comprehensive plant‐part‐stratified individual patient data meta‐analysis or systematic review of adverse events across this trial landscape would constitute a substantial and independent undertaking, beyond the regulatory‐focused scope of the present narrative review. Such a review would be both methodologically robust and directly policy‐informing, and is identified here as a priority research need. Notably, even systematic reviews that incidentally include both root‐only and root‐and‐leaf preparations, such as the sleep meta‐analysis by (Cheah et al. 2021), do not conduct plant‐part‐stratified safety analyses, further underscoring the absence of the comparative evidence base that would be required to draw definitive conclusions about differential adverse event profiles between product types.

In this context, future research strategies should move explicitly towards plant‐part‐specific science and governance. Scientifically, there is a clear need for: (i) rigorously designed, comparative RCTs that evaluate authenticated root, leaf, and whole‐plant extracts with matched chemotyping and withanolide profiles in discrete indications; (ii) comprehensive reproductive, developmental, hepatic, and thyroid safety studies for leaf and high‐withaferin A preparations, with robust dose–response characterization; (iii) mechanistic work to define human therapeutic and toxic windows for withaferin A; and (iv) a dedicated systematic review and, where data permit, individual patient data meta‐analysis of adverse events reported across all published clinical trials of *W. somnifera* , stratified explicitly by plant part (root‐only versus root‐and‐leaf versus whole‐plant), dose, withaferin A content, and trial duration. Such a plant‐part‐stratified safety synthesis is currently absent from the literature and would provide the evidentiary foundation necessary for proportionate, evidence‐based regulatory decision‐making across jurisdictions.

From a regulatory standpoint, monographs and guidance should define “ashwagandha” for internal therapeutic or supplement use as root or root extract unless leaf/aerial parts are explicitly declared; separate monographs and dossiers should be developed for leaf or whole‐plant products, with their own toxicological and clinical evidence base. Regulators should require validated orthogonal methods to distinguish root from leaves/stems as part of GMP release and market‐surveillance testing and treat undeclared aerial‐part content in root‐labeled products as adulteration and misbranding, with appropriate sanctions. A scientifically sound regulatory framework for *WS* leaf should, in principle, incorporate a dose‐based approach anchored to quantification and limitation of withaferin A as the principal toxicologically relevant constituent. Withaferin A exhibits a well‐documented biphasic dose–response profile: at low concentrations it demonstrates anti‐inflammatory and cytoprotective effects, whereas at higher exposures it exerts cytotoxic and genotoxic activity in preclinical systems (Widodo et al. 2008; Dutta et al. 2019). This concentration‐dependence is pharmacologically important and analogous, in principle, to regulatory thresholds established for other potent botanical constituents such as pyrrolizidine alkaloids, where EMA and EFSA have defined maximum daily intake limits based on cumulative toxicological datasets (Berra et al. 2024; ANSES 2024). However, a critical prerequisite for setting a safe withaferin A threshold is currently absent: there are no adequately powered, long‐term human pharmacokinetic or dose‐escalation studies for leaf‐derived preparations that would permit derivation of a NOAEL or Benchmark Dose in healthy populations. Existing Phase I data in osteosarcoma patients (Pires et al. 2020) cannot be extrapolated to general supplement users. On current evidence, a precautionary position would restrict leaf‐containing preparations from the general supplement market pending dose–response data, while noting that this restriction is provisional and revisable as leaf‐specific safety data emerge. Risk assessments, particularly in Europe, should stratify evidence by plant part, dose, duration, and product type, and re‐evaluate earlier highly precautionary opinions that were based on plant‐part‐agnostic data aggregation, with the aim of restoring proportionality and ensuring continued access to evidence‐based root preparations while maintaining justified precautions in vulnerable populations.

Clear labeling of plant part, extraction ratio, standardization markers, and withaferin A content would further enable clinicians, regulators, and consumers to align product selection with the distinct risk–benefit profiles of root and leaf. In sum, the available data justify a regulatory stance that recognizes ashwagandha root as the reference standard for internal use, while encouraging carefully controlled, indication‐specific development of leaf‐based preparations and closing the gap between scientific differentiation and regulatory practice.

## 5 Conclusion

This review concludes that ashwagandha safety and efficacy are fundamentally plant‐part dependent, and that authenticated root material is currently the best‐characterized material for systemic internal use and is supported by the available evidence as the reference standard. Traditional use, phytochemical gradients, multiple randomized controlled trials, a 12‐month safety study, and AI‐assisted toxicology collectively support a favorable benefit–risk profile for standardized root extracts within the studied dose ranges. In contrast, leaf and whole‐plant preparations are richer in withaferin A, underlie much of the experimental anticancer and immunomodulatory literature, have sparse and heterogeneous clinical data, and are disproportionately implicated in toxicological discussions, especially where products are adulterated or mislabeled. Regulatory experiences in Denmark and other European states illustrate that plant‐part‐agnostic risk assessments can lead to over‐precautionary bans that are misaligned with root‐specific evidence. Regulators should therefore codify plant‐part specificity, mandate robust authentication to detect leaf adulteration, stratify risk assessments by plant part and product type, and prioritize comparative trials and long‐term leaf safety studies to enable proportionate, evidence‐based governance. Until adequately powered safety data for leaf preparations become available, the current evidence provides a stronger basis for the use of standardized root products in systemic use and supports regulatory attention to the authentication and nonadulteration of root materials. This position is precautionary and should be revisited as leaf‐specific evidence develops.

## Author Contributions

**Abhijit Dutta:** conceptualization, methodology, data curation, investigation, validation, formal analysis, supervision, project administration, resources, writing – original draft, writing – review and editing, software, visualization. **Jang Bahadur Gupta:** conceptualization, investigation, validation, supervision, data curation, writing – review and editing.

## Funding

The authors have nothing to report.

## Disclosure

Declaration on the Use of AI: No generative AI was used during the preparation of this article.

## Ethics Statement

Ethics approval is not applicable as this study used secondary data from published studies.

## Conflicts of Interest

The authors declare no conflicts of interest. The views expressed in this article are based on the evidence and data reviewed by the authors and represent their views in their personal capacities. They do not necessarily represent the official position of their affiliated organizations or the Government of India.

## Supporting information


**Table S1:** Electronic database search strategy.
**Table S2:** Gray‐literature and official/regulatory sources.

## Data Availability

The data that support the findings of this study are available from the corresponding author upon reasonable request.

## References

[fsn372364-bib-0001] Akhgarjand, C. , F. Asoudeh , A. Bagheri , et al. 2022. “Does Ashwagandha Supplementation Have a Beneficial Effect on the Management of Anxiety and Stress? A Systematic Review and Meta‐Analysis of Randomized Controlled Trials.” Phytotherapy Research 36: 4115–4124. 10.1002/ptr.7598. 36017529

[fsn372364-bib-0002] Amritha, N. , V. Bhooma , and M. Parani . 2020. “Authentication of the Market Samples of Ashwagandha by DNA Barcoding Reveals That Powders Are Significantly More Adulterated Than Roots.” Journal of Ethnopharmacology 256: 112725. 10.1016/j.jep.2020.112725. 32126246

[fsn372364-bib-0003] ANSES . 2024. “AVIS de l'Anses relatif aux risques liés à l'utilisation des préparations de Withania somnifera (L.) Dunal dans les compléments alimentaires.”

[fsn372364-bib-0004] Arumugam, V. , V. Vijayakumar , A. Balakrishnan , et al. 2024. “Effects of Ashwagandha ( *Withania somnifera* ) on Stress and Anxiety: A Systematic Review and Meta‐Analysis.” Explore (New York, N.Y.) 20: 103062. 10.1016/j.explore.2024.103062. 39348746

[fsn372364-bib-0005] Auddy, B. , J. Hazra , A. Mitra , B. Abedon , and S. Ghosal . 2008. “A Standardized *Withania somnifera* Extract Significantly Reduces Stress‐Related Parameters in Chronically Stressed Humans: A Double‐Blind, Randomized, Placebo‐Controlled Study.” Journal of the American Nutraceutical Association 11: 50–56.

[fsn372364-bib-0006] Bashir, A. , M. Nabi , N. Tabassum , S. Afzal , and M. Ayoub . 2023. “An Updated Review on Phytochemistry and Molecular Targets of *Withania somnifera* (L.) Dunal (Ashwagandha).” Frontiers in Pharmacology 14: 1049334. 10.3389/fphar.2023.1049334. PMC10090468 37063285

[fsn372364-bib-0007] Beladiya, U. , J. Chaliha , V. A. Mevada , H. Gandhi , and R. K. Patel . 2024. “Healing Through Herbs: Everaging Herbal Remedies Against COVID‐19.” In Herb. Formul. Phytochem. Pharmacogn, 343–359. Elsevier. 10.1016/B978-0-443-15383-9.00015-9.

[fsn372364-bib-0008] Berra, J. L. , S. Chernigoy , K. Alvarez , and M. J. Wernisch . 2024. “Ashwagandha Under Fire: A Critical Scientific Analysis of Regulatory Decisions.” Int J Ayurveda Res 5: 148–153. 10.4103/ijar.ijar_192_24.

[fsn372364-bib-0009] BfR . 2024. “Ashwagandha: Food Supplements With Potential Health Risks.”

[fsn372364-bib-0010] Bhosale, S. D. , G. Ruknuddin , and P. K. Prajapati . 2022. “Can Ashwagandha Leaf Be Replaced With Its Root in Therapeutics? A Review Through Published Literature.” Pharmacognosy Research 15: 1–12. 10.5530/097484900286.

[fsn372364-bib-0011] Bokan, G. , T. Glamočanin , Z. Mavija , et al. 2023. “Herb‐Induced Liver Injury by Ayurvedic Ashwagandha as Assessed for Causality by the Updated RUCAM: An Emerging Cause.” Pharmaceuticals 16: 1129. 10.3390/ph16081129. PMC10459262 37631044

[fsn372364-bib-0012] Brendler, T. , R. Al‐Mondhiry , L. Lang , R. Marles , M. Tallon , and A. Raghu . n.d. “Ashwagandha: Is It Safe? Part 1: A Regulatory Review.” 10.1002/ptr.70151. PMC13436186 41420287

[fsn372364-bib-0013] Chaurasiya, N. D. , G. C. Uniyal , P. Lal , et al. 2008. “Analysis of Withanolides in Root and Leaf of *Withania somnifera* by HPLC With Photodiode Array and Evaporative Light Scattering Detection.” Phytochemical Analysis 19: 148–154. 10.1002/pca.1029. 17879227

[fsn372364-bib-0014] Cheah, K. L. , M. N. Norhayati , L. Husniati Yaacob , and R. Abdul Rahman . 2021. “Effect of Ashwagandha ( *Withania somnifera* ) Extract on Sleep: A Systematic Review and Meta‐Analysis.” PLoS One 16: e0257843. 10.1371/journal.pone.0257843. PMC8462692 34559859

[fsn372364-bib-0015] Chittiboyina, A. G. , and I. A. Khan . 2024. “Current Issues in Phytomedicine Research ‐ Conundrum on the Chemistry of Ashwagandha and Its Biological Effects.” Journal of Ethnopharmacology 325: 117871. 10.1016/j.jep.2024.117871. 38325672

[fsn372364-bib-0016] Dar, N. J. , A. Hamid , and M. Ahmad . 2015. “Pharmacologic Overview of *Withania somnifera* , the Indian Ginseng.” Cellular and Molecular Life Sciences 72: 4445–4460. 10.1007/s00018-015-2012-1. PMC11113996 26306935

[fsn372364-bib-0017] Della Porta, M. , J. A. Maier , and R. Cazzola . 2023. “Effects of *Withania somnifera* on Cortisol Levels in Stressed Human Subjects: A Systematic Review.” Nutrients 15: 5015. 10.3390/nu15245015. PMC10745833 38140274

[fsn372364-bib-0018] Dutta, R. , R. Khalil , R. Green , S. S. Mohapatra , and S. Mohapatra . 2019. “ *Withania somnifera* (Ashwagandha) and Withaferin A: Potential in Integrative Oncology.” International Journal of Molecular Sciences 20: 5310. 10.3390/ijms20215310. PMC6862083 31731424

[fsn372364-bib-0019] EMA . 2011. “Withaniae Somniferae Radix ‐ Herbal Medicinal Product | European Medicines Agency (EMA).” https://www.ema.europa.eu/en/medicines/herbal/withaniae‐somniferae‐radix.

[fsn372364-bib-0020] Fatima, K. , J. Malik , F. Muskan , et al. 2024. “Safety and Efficacy of *Withania somnifera* for Anxiety and Insomnia: Systematic Review and Meta‐Analysis.” Human Psychopharmacology: Clinical and Experimental 39: e2911. 10.1002/hup.2911. 39083548

[fsn372364-bib-0021] FSA . 2024. “Ashwagandha | Food Standards Agency.” https://www.food.gov.uk/news‐alerts/consultations/ashwagandha.

[fsn372364-bib-0022] Gómez Afonso, A. , D. Fernandez‐Lazaro , D. P. Adams , A. Monserdà‐Vilaró , and C. I. Fernandez‐Lazaro . 2023. “Effects of *Withania somnifera* (Ashwagandha) on Hematological and Biochemical Markers, Hormonal Behavior, and Oxidant Response in Healthy Adults: A Systematic Review.” Current Nutrition Reports 12: 465–477. 10.1007/s13668-023-00481-0. PMC10444651 37428341

[fsn372364-bib-0023] Guo, S. , and M. J. Rezaei . 2024. “The Benefits of Ashwagandha ( *Withania somnifera* ) Supplements on Brain Function and Sports Performance.” Frontiers in Nutrition 11: 1439294. 10.3389/fnut.2024.1439294. PMC11327513 39155932

[fsn372364-bib-0024] Heo, J. U. , S. Rao , H. B. Newton , A. Dowlati , R. F. Muzic , and A. Kardan . 2026. “Phase 1 Trial of *Withania somnifera* Leaf Extract (RH324) in Advanced Non‐Small Cell Lung Cancer Including [18F]FDG PET/CT as a Short‐Term Metabolic Biomarker to Assess Efficacy: A Novel Model for Assessment of Complimentary Therapies in Early Phase Human Clinical Trials.” Integrative Cancer Therapies 25: 15347354251410182. 10.1177/15347354251410182. PMC12905094 41685666

[fsn372364-bib-0025] Kaul, S. , and R. Wadhwa . 2024. Ashwagandha for Quality of Life: Scientific Evidence. CRC Press (Taylor & Francis Group).

[fsn372364-bib-0026] Kaul, S. C. , and R. Wadhwa , eds. 2017. Science of Ashwagandha: Preventive and Therapeutic Potentials. Springer International Publishing. 10.1007/978-3-319-59192-6.

[fsn372364-bib-0027] Khabiya, R. , G. P. Choudhary , A. C. Jnanesha , A. Kumar , and R. K. Lal . 2024. “An Insight Into the Potential Varieties of Ashwagandha (Indian Ginseng) for Better Therapeutic Efficacy.” Ecological Frontiers 44: 444–450. 10.1016/j.chnaes.2023.06.009.

[fsn372364-bib-0028] Kumar, A. , B. K. Sah , F. Ahmad , V. Jamwal , and S. K. Sahu . 2026. “Potential Adverse Effects of Ashwagandha: A Critical Review of Preclinical and Clinical Evidence.” Phytotherapy Research 40: 4913–4931. 10.1002/ptr.70158. PMC13436050 41502355

[fsn372364-bib-0029] Kumar, B. , V. Bajpai , V. Gond , S. Tiwari , and K. P. Madhusudanan . 2024. Phytochemistry of *Withania somnifera* . CRC Press (Taylor & Francis Group).

[fsn372364-bib-0030] Kumar, P. , S. P. Banik , A. Goel , S. Chakraborty , M. Bagchi , and D. Bagchi . 2023. “A Critical Assessment of the Whole Plant‐Based Phytotherapeutics From *Withania somnifera* (L.) Dunal With Respect to Safety and Efficacy Vis‐a‐Vis Leaf or Root Extract‐Based Formulation.” Toxicology Mechanisms and Methods 33: 698–706. 10.1080/15376516.2023.2242933. 37533233

[fsn372364-bib-0031] Lopresti, A. L. , and S. J. Smith . 2021. “Ashwagandha ( *Withania somnifera* ) for the Treatment and Enhancement of Mental and Physical Conditions: A Systematic Review of Human Trials.” Journal of Herbal Medicine 28: 100434. 10.1016/j.hermed.2021.100434.

[fsn372364-bib-0032] Lucius, K. 2025. “Clinical Evidence for the Use of Ashwagandha, *Withania somnifera* .” Integrative and Complementary Therapies 31: 54–62. 10.1089/ict.2025.28106.kl.

[fsn372364-bib-0033] Mikulska, P. , M. Malinowska , M. Ignacyk , et al. 2023. “Ashwagandha ( *Withania somnifera* )—Current Research on the Health‐Promoting Activities: A Narrative Review.” Pharmaceutics 15: 1057. 10.3390/pharmaceutics15041057. PMC10147008 37111543

[fsn372364-bib-0034] MoA . 2024a. Safety of Ashwagandha ( *Withania somnifera* ) Report of the Expert Committee Constituted by Ministry of Ayush, Govt. of India. Central Council for Research in Ayuredic Sciences.

[fsn372364-bib-0035] MoA . 2024b. “Ashwagandha ‐ Safety Dossier 2.0.”

[fsn372364-bib-0036] Morandi, A. 2024. “When Regulation Ignores Evidence: The Case of Denmark's Ashwagandha Ban and the Technical University of Denmark's Misguided Risk Assessment.” Int J Ayurveda Res 5: 144–147. 10.4103/ijar.ijar_171_24.

[fsn372364-bib-0037] NIDDK . 2012. “Ashwagandha.” In LiverTox Clin. Res. Inf. Drug‐Induc. Liver Inj. National Institute of Diabetes and Digestive and Kidney Diseases.

[fsn372364-bib-0038] Pandit, S. , A. K. Srivastav , T. K. Sur , S. Chaudhuri , Y. Wang , and T. K. Biswas . 2024. “Effects of *Withania somnifera* Extract in Chronically Stressed Adults: A Randomized Controlled Trial.” Nutrients 16: 1293. 10.3390/nu16091293. PMC11085552 38732539

[fsn372364-bib-0039] Patwardhan, B. , S. Chaturvedi , G. Tillu , S. Deshpande , and B. M. Hegde . 2024. “Danish Ban on Ashwagandha: Truth, Evidence, Ethics, and Regulations.” J Ayurveda Integr Med 15: 101028. 10.1016/j.jaim.2024.101028. PMC11403136 38969606

[fsn372364-bib-0040] Pires, N. , V. Gota , A. Gulia , L. Hingorani , M. Agarwal , and A. Puri . 2020. “Safety and Pharmacokinetics of Withaferin‐A in Advanced Stage High Grade Osteosarcoma: A Phase I Trial.” J Ayurveda Integr Med 11: 68–72. 10.1016/j.jaim.2018.12.008. PMC7125369 30904387

[fsn372364-bib-0041] RIVM . 2024. Risk Assessment of Herbal Preparations Containing *Withania somnifera* (Ashwagandha). Rijksinstituut voor Volksgezondheid en Milieu RIVM. 10.21945/RIVM-2024-0029.

[fsn372364-bib-0042] Singh, A. , M. Ali , D. K. Mishra , and S. Kanojiya . 2025. “Comparative Profiling of Withanolides in Tissues of Withania Somnifera and *Datura metel* Using UHPLC‐MS/MS.” Chemistry & Biodiversity 22: e01319. 10.1002/cbdv.202501319. 40845291

[fsn372364-bib-0043] Singh, N. , M. Bhalla , P. de Jager , and M. Gilca . 2011. “An Overview on Ashwagandha: A Rasayana (Rejuvenator) of Ayurveda.” African Journal of Traditional, Complementary, and Alternative Medicines 8: 208–213. 10.4314/ajtcam.v8i5S.9. PMC3252722 22754076

[fsn372364-bib-0044] Singh, V. K. , D. Mundkinajeddu , A. Agarwal , et al. 2018. Adulteration of Ashwagandha (Withania somnifera) Roots and Extracts. Botanical Adulterants Prevention Bulletin. ABC‐AHP‐NCNPR Botanical Adulterants Prevention Program. https://www.herbalgram.org/resources/botanical‐adulterants‐prevention‐program/adulterants‐bulletins/ashwagandha‐bulletin‐january‐2019/.

[fsn372364-bib-0045] Singirala, S. K. , P. K. Dubey , and S. Roy . 2025. “Extraction of Bioactive Compounds From *Withania somnifera* : The Biological Activities and Potential Application in the Food Industry: A Review.” International Journal of Food Science 2025: 9922626. 10.1155/ijfo/9922626. PMC12048197 40322538

[fsn372364-bib-0046] Tandon, N. , and S. S. Yadav . 2020. “Safety and Clinical Effectiveness of *Withania somnifera* (Linn.) Dunal Root in Human Ailments.” Journal of Ethnopharmacology 255: 112768. 10.1016/j.jep.2020.112768. 32201301

[fsn372364-bib-0047] Technical University of Denmark (DTU) . 2020. “Risk Assessment of the Root of *Withania somnifera*.”

[fsn372364-bib-0048] Thorat, S. A. , A. Kaniyassery , P. Poojari , et al. 2022. “Differential Gene Expression and Withanolides Biosynthesis During In Vitro and Ex Vitro Growth of *Withania somnifera* (L.) Dunal.” Frontiers in Plant Science 13: 917770. 10.3389/fpls.2022.917770. PMC9237602 35774803

[fsn372364-bib-0049] Tsoulli, C. , S. Thomas , and C. Potter . 2025. “P26‐04 Review of the Safety of Ashwagandha ( *Withania somnifera* (L.) Dunal) in Food, Drinks and Food Supplements.” Toxicology Letters 411: S326–S326a. 10.1016/j.toxlet.2025.07.758.

[fsn372364-bib-0050] Wadhwa, R. , A. Konar , and S. C. Kaul . 2016. “Nootropic Potential of Ashwagandha Leaves: Beyond Traditional Root Extracts.” Neurochemistry International 95: 109–118. 10.1016/j.neuint.2015.09.001. 26361721

[fsn372364-bib-0051] Wadhwa, R. , R. Singh , R. Gao , et al. 2013. “Water Extract of Ashwagandha Leaves Has Anticancer Activity: Identification of an Active Component and Its Mechanism of Action.” PLoS One 8: e77189. 10.1371/journal.pone.0077189. PMC3795014 24130852

[fsn372364-bib-0052] Wiciński, M. , A. Fajkiel‐Madajczyk , J. Sławatycki , et al. 2025. “Ashwagandha ( *Withania somnifera* ) and Its Effects on Well‐Being—A Review.” Nutrients 17: 2143. 10.3390/nu17132143. PMC12252077 40647248

[fsn372364-bib-0053] Widodo, N. , Y. Takagi , B. G. Shrestha , T. Ishii , S. C. Kaul , and R. Wadhwa . 2008. “Selective Killing of Cancer Cells by Leaf Extract of Ashwagandha: Components, Activity and Pathway Analyses.” Cancer Letters 262: 37–47. 10.1016/j.canlet.2007.11.037. 18191020

[fsn372364-bib-0054] Zellner, L. , M. Wierer , M. Himmelsbach , B. Schatzmann , B. Thalhamer , and C. W. Klampfl . n.d. “Determination of Withanolides and Withanosides in Ashwagandha Based Products Using HPLC‐Drift‐Tube‐Ion‐Mobility Quadrupole Time‐of‐Flight Mass Spectrometry.” 10.1002/elps.202400188. PMC11952284 39965076

[fsn372364-bib-0055] Ziegenfuss, T. N. , A. W. Kedia , J. E. Sandrock , B. J. Raub , C. M. Kerksick , and H. L. Lopez . 2018. “Effects of an Aqueous Extract of *Withania somnifera* on Strength Training Adaptations and Recovery: The STAR Trial.” Nutrients 10: 1807. 10.3390/nu10111807. PMC6266766 30463324

