# Supplementary material for: Plant Part–Specific Differences in Ashwagandha (Withania somnifera): A Comparative Evaluation of Root and Leaf Evidence for Safety and Regulation
Source: Food Sci Nutr. 2026 Oct 5;14(10):e72364. doi: 10.1002/fsn3.72364 (PMC13635981; doi:10.1002/fsn3.72364)
Supplement: Supplementary file 1 [file FSN3-14-e72364-s001.docx]

**Supplementary-1**

**Table A. Electronic database search strategy**

| **Database** | **Platform / interface** | **Field tags searched** | **Search string (Boolean)** | **# of Hits** | **Date of search** |
| --- | --- | --- | --- | --- | --- |
| PubMed | NCBI | Title/Abstract, MeSH | ("Withania somnifera" OR Ashwagandha) AND (root OR leaf OR "aerial parts" OR withanolides OR withaferin) AND (safety OR toxicity OR hepatotoxicity OR regulatory OR adulteration OR efficacy) | 445 | 31 March 2026 |
| Scopus | Elsevier | TITLE-ABS-KEY | TITLE-ABS-KEY(("Withania somnifera" OR Ashwagandha) AND (root OR leaf OR "aerial parts" OR withanolides) AND (safety OR toxicity OR regulatory OR adulteration)) | 523 | 31 March 2026 |
| Web of Science | Clarivate (Core Collection) | Topic (TS) | TS=(("Withania somnifera" OR Ashwagandha) AND (root OR leaf OR "aerial parts" OR withanolides) AND (safety OR toxicity OR regulatory OR adulteration OR Europe OR DTU)) | 329 | 31 March 2026 |
| Google Scholar | Google | Full text / title | "Withania somnifera" OR Ashwagandha root leaf safety toxicity regulatory adulteration | First 200 records screened by relevance ranking | 31 March 2026 |

Note: *Searches were supplemented by backward and forward citation tracking (snowballing) of key reviews and regulatory safety evaluations. Search terms were combined with Boolean operators; where database syntax differed, strings were adapted while preserving the core concept blocks (plant identity × plant part/constituent × safety/regulatory outcome).*

**Table B. Grey-literature and official / regulatory sources**

| **Source** | **Body / jurisdiction** | **Type of document** | **Relevance to review** |
| --- | --- | --- | --- |
| Ministry of Ayush (AYUSH) | Government of India | Expert committee report; Safety Dossier 2.0 | Root safety evidence; plant-part position; critique of EU assessments |
| DTU National Food Institute | Denmark (Technical University of Denmark) | Risk assessment (2020) | Basis for Danish supplement ban; plant-part-agnostic assessment |
| RIVM | Netherlands | Risk assessment (2024) | Hepatotoxicity/endocrine signals; "do not use" advice |
| BfR | Germany (Federal Institute for Risk Assessment) | Risk opinion (2024) | Hepatotoxicity, thyroid, reproductive concerns |
| ANSES | France | Risk assessment (avis, 2024) | Safety concerns; calls for dedicated studies |
| UK Food Standards Agency (FSA) | United Kingdom | Consultation / safety review (2024) | Plant-part specification; labelling |
| EMA | European Union | Herbal monograph — *Withaniae somniferae radix* (2011) | Root-based pharmacopoeial reference |
| WHO | International | Herbal medicine classification / monographs | Global regulatory categorization |
| Botanical Adulterants Prevention Program (BAPP) | United States (ABC-AHP-NCNPR) | Bulletin + Laboratory Guidance Document | Root adulteration with leaf/stem; analytical authentication |

Note: *Grey-literature sources were identified through targeted searching of official agency websites and citation tracking. Only documents explicitly addressing Withania somnifera plant parts, safety, quality, or regulatory status were retained.*
